# A Study of Lac-Caninum

**Published:** 1891-02

**Authors:** D. C. Perkins

**Affiliations:** Rockland, Maine


### A STUDY OF LAC CANINUM.

D. C. PERKINS, M. D., ROCKLAND, MAINE.

(A paper read before the Maine Homœopathic Medical Society, and published in its transactions for 1890.)

It sometimes happens that physicians as well as other worthy people allow their minds to be influenced by prejudice. It might be said that our brethren of allopathic proclivities are in a chronic condition of prejudice toward the truths and virtues of that law of cure discovered by Samuel Hahnemann and denominated Homœopathy. But the weakness of prejudice is not wholly limited to allopathic ranks. There are cases in our own school which are unaccountable, unreasonable, and to common minds, unjustifiable. Who has not heard, or read, denunciations of some of our polychrests even, as being unworthy the proof of being proved. Lachesis, Lycopodium, Natrum muriaticum, and others equally as well known have each in turn been under the ban of condemnation by a strong percentage of professed homœopathists. Just now the remedy which is being pooh-poohed is Lac caninum, whose medical virtues, those who have tested it have no reason to doubt. It being a strong and far-reaching remedy, worthy of study, confidence, and use, has induced me to prepare and present this paper.

*Mind.*—Forgetful, nervous, very restless, cannot bear to be left alone; depression of spirits; believes he is fatally sick. Unable to concentrate the mind. Wants to leave everything as soon as it is commenced. Gloomy and apprehensive. Cross and irritable. Intense ugliness and hatefulness.

*Head.*—Constant noise in head, very confusing, worse at night, and at time of menses. Wakes at night with sensation as if the bed was in motion. Headache, aggravated by noise and talking, relieved by keeping quiet. Unbearable pains in head, change from one side to the other. Headache from below eyes over whole head. Head very sore and itches almost all the time. Sore pimples on scalp which discharge and form a scab; extremely painful when touched, or when combing hair.

*Eyes and Sight.*—Eyes sensitive to light, must have light, yet intolerant of sunlight. Retina retains impressions of subjects, especially of colors; film over eyes from reading. Eyes watery, dull, and lustreless. Upper eyelids heavy.

*Ears and Hearing.*—Sounds seem far off. Pain in both ears; noises as if ears were full. Deafness from hereditary syphilis.

*Nose and Smell.*—Nose cold. Fluids escape through nose while drinking. Nose stuffed, obstructing breathing; coryza with discharge of thick white mucus; upper part of nose seems full.

*Face.*—Face flushed; cheeks red. Lips dry, peeling off; dry and parched, but mouth constantly full of tough, frothy saliva.

*Mouth.*—Putrid taste in mouth. Tongue coated, whitish or dirty looking. Indistinct utterance. Breath offensive, putrid.

*Throat.*—Throat very sensitive externally. Breathing arrested on going to sleep. Sensation as if throat were closing.

Paralytic symptoms strongly marked; swallowing difficult, painful, almost impossible. Pricking sensation in throat as if full of sticks; uvula elongated, greatly swollen; feeling of lump in throat; goes down when swallowing, but returns again; shining, glazed, red appearance of throat; soreness of throat, commences with a tickling sensation which causes cough; *tonsils inflamed and very sore, red and shining, almost closing the throat. Whole membrane of throat swollen, dark red, with gray patches and small irregular shaped ulcers. Whole membrane of throat highly inflamed, swollen, and glands enlarged on both sides. The membrane is thick, yellowish gray, often greenish.*

These symptoms of the throat are but a few of those produced by this remedy, and when the whole are considered, so striking a picture of diphtheria is presented that in many cases to refuse to prescribe it would be to ignore the homœopathic law.

*Appetite and Stomach.*—Appetite and strength failing; no appetite; dyspepsia; thirst produced by dryness of throat; nausea with headache on waking; burning in epigastric region.

*Abdomen.*—Abdomen very hard and swollen in evening; pressure from within outward in lower abdomen. Pain in pelvis, principally in right ovarian region; pains in abdomen, intermittent.

*Urinary Organs.*—Constant desire to urinate with intense pain; urine unusually frequent and dark; great difficulty in urinating.

*Voice, Respiration, Chest, etc.*—Unable to speak aloud; distressed feeling while speaking; excessive hoarseness and tickling sensation, better when moving about; breathing hoarse and croupy, at times entire stoppage of breath. Cough from tickling in upper anterior part of larynx; worse from talking or lying down; hard metallic cough; croupy cough; sharp, incisive pains between scapulæ, passing through to sternum; trembling, jerking, and fluttering through lungs.

Pulse quick, full, and strong, with pain in chest and throat.

*Lactation.*—(Serviceable in almost all cases where it is required to dry up milk.)

*Neck, Back, and Limbs.*—Neck stiff; pain in back of neck; spine aches from base of brain to coccyx; heat, pain, and beating in small of back; shoulders and arms ache; almost constant pain in right hip. Articular rheumatism in right hip and knee-joints, especially the former; intense unbearable pain across supra sacral region extending to right natis and down right sciatic nerve; bruised pain in soles of feet, with stiffness of ankle, knee, and hip joints; numb pains chiefly in ankles; pain in limbs as if beaten.

*Nerves.*—Profound depression of vitality. *General weakness and prostration very marked; sinking spells every morning, attended with great nervousness.* When walking, seems to be walking on air; when lying does not seem to touch the bed.

*Sensations.*—Throat feels full of sticks, or as if scalded by hot fluid; pain as from a stone in pit of stomach; pain over eyes, in temples, in both ears, in whole body and limbs; in right thigh and uterus; in back of neck; in nipples, chest, and throat. The pains which attack different parts are mentioned as violent, intense, sharp, severe, lancinating, cutting, stabbing, darting, piercing, beating, acute, terrible, excruciating, unbearable, showing that the conditions demanding this remedy are as intense as those calling for Arsenicum.

Almost every region is affected. We have symptoms relating to forehead, top and back of head, brain, eyeballs, neck, cheeks, ears, nose, mouth, tongue, lips, throat, chest, heart, stomach, bowels, kidneys, liver, spleen, urinary and sexual organs, back, joints, arms, legs, wrists, ankles, fingers, toes, hearing, sight, taste, and smell.

Lac-caninum is related in its pathogenetic effects, and of course in its curative action, to a large number of remedies. Among these prominently are Arsenicum, Hepar, Belladonna, Lachesis, Graphites, Lycopodium, Kali-bich., Natrum-muriaticum. In a less degree to Anacardium, Apis-mel., Aconite, Bovista, Bryonia, Calcarea-carb., Causticum, Dulcamara, Eupatorium, Gnaphalium, Gelsemium, Phosphorus, Ruta, Sanguinaria, Stramonium, Thuja, Psorinum, and Sulphur.

Thus, briefly, are presented some of the leading symptoms and relations of Lac-caninum, a remedy which is destined to occupy a place in therapeutics not less prominent than Carbo-vegetabilis, Lachesis, or Veratrum. Its adaptability is not limited by age, sex, color, temperament, or unbelief in its curative properties. Most heartily I commend it to my colleagues, in the firm conviction that it will fully meet their highest expectations.

